# Neck stabilization exercise and dynamic neuromuscular stabilization reduce pain intensity, forward head angle and muscle activity of employees with chronic non‐specific neck pain: A retrospective study

**DOI:** 10.1002/jeo2.70188

**Published:** 2025-02-28

**Authors:** Ainollah Sakinepoor, Zahra Ataei Cheragh, Hans Degens, Maryam Mazidi

**Affiliations:** ^1^ Department of Physical Education Tehran Iran; ^2^ Sports Injury and Corrective Exercises, Faculty of Physical Education and Sports Sciences Razi University Kermanshah Iran; ^3^ Department of Life Sciences Research Centre for Musculoskeletal Science & Sports Medicine Manchester Metropolitan University Manchester UK; ^4^ Institute of Sport Science and Innovations Lithuanian Sports University Kaunas Lithuania; ^5^ Sport Injury and Corrective Exercises Hormozgan University Bandarabbas Iran

**Keywords:** chronic non‐specific neck pain, dynamic neuromuscular stabilization exercise, electromyography, forward head posture, Numeric Pain Rating Scale, stabilization exercise

## Abstract

**Purpose:**

Previous investigations have associated weakness of neck muscles with a higher likelihood of developing neck pain. However, no previous investigation has examined the influence of neck stabilization exercise (NSE) and dynamic neuromuscular stabilization (DNS) on pain intensity, forward head angle (FHA) and muscle activity.

**Methods:**

A total of 45 female employees with chronic non‐specific neck pain (CNNP) underwent measurements of pain intensity, FHA and electrical activity of muscles in a slump posture, before and after either NSE or DNS.

**Results:**

After both stabilization exercise (SE) and DNS the Numeric Pain Rating Scale (NPRS) (*F* (2,39) = 17.61, *p* = 0.001, partial *η*² = 0.475) and forward head posture (FHP), (*F* (2,39) = 5.509, *p* = 0.008, partial *η*² = 0.220), had decreased. Both interventions also decreased the activity in the cervical erector spinae muscle (*F* (2,39) = 5.31, *p* = 0.009, partial *η*² = 0.214), the upper trapezius muscle (*F* (2,39) = 5.41, *p* = 0.008, partial *η*² = 0.217) in slump typing posture, but there was no significant effect on the activity in the sternocleidomastoid muscle (*F* (2,39) = 2.65, *p* = 0.083, partial *η*² = 0.120).

**Conclusion:**

Both DNS and SE exercises diminished pain intensity, forward head and muscle activity after 6 weeks in patients with CNSNP.

**Level of Evidence:**

Level I, randomized controlled trials with adequate statistical power.

**Abbreviations**
CES: cervical erector spine
CNNP: chronic non‐specific neck pain
DNS: dynamic neuromuscular stabilization
EMG: electromyography
FHP: forward head posture
MVC: maximal voluntary contraction
NPRS: Numeric Pain Rating Scale
NSE: neck stabilization exercise
SCM: sternocleidomastoid
UT: upper trapezius

## INTRODUCTION

Working is an important part of human life but it can lead to work‐related musculoskeletal disorders [54, 55, 63], one of the most frequent occupational health problems, especially in developing countries [4]. Neck pain is a common musculoskeletal complication in modern society, and the second most prevalent musculoskeletal disorder after back pain [25]. Often this pain displays itself with a feeling of discomfort between the lower edge of the occipital bone and the first thoracic vertebra [51], Neck pain is often chronic and its cause is unknown in most cases [45].

The increased prevalence of neck pain in the last two decades and disability among these individuals pose an economic and social burden for the individual and society [66]. In fact, 42%–69% of office workers have reported at least one case of neck pain during their working life [41].

Office workers, while working on the computer or doing other office work behind the desk, are in a cycle of repetitive movements, which in the long run can cause the person to adopt a wrong sitting pattern. The person herself/himself may not even be aware of the physical posture while working, such as the position of the head forward, which can put the anti‐gravity neck muscle group of these people in a state of strain and fatigue [3, 24, 37, 68]. The forward head posture (FHP), by moving the centre of gravity forward, leads to long‐term stretching of the neck and shoulder muscles that stabilize the head when the neck is bent [35, 51]. A persistent FHP not only imposes a heavy burden on the muscles, but also on the spine, which adds to the challenge to the muscles of the neck and upper limbs [52, 64].

In previous studies, neck muscle weakness has been reported in individuals with neck pain compared to healthy people. This is perhaps attributable to a disturbed coordination between the deep and superficial flexor muscles of the neck, where the electrical activity of sterno‐pectoral muscles acting as surface flexors is increased and the electrical activity of longus colli and longus capitis muscles, deep flexors of the neck, is decreased [49]. This altered muscle recruitment in the head forward position for extended periods may well result in muscle weakness and fatigue often seen in patients with neck pain [27].

Although the number of people seeking treatment for neck pain is significant, there is no clear and successful treatment, and research in this field is limited. The goal of effective treatment is not only to alleviate neck pain and clinical symptoms but also to prevent their recurrence [16, 17].

Various interventions such as manual therapy, medication, heat, and exercise have been used to improve symptoms in individuals with non‐specific chronic neck pain [19, 30, 41, 67]. Although physical therapy not only improves the quality of life and reduces pain but also increases strength and endurance of neck muscles, stabilizing exercise (SE) and dynamic neuromuscular stabilization (DNS) have shown to be more effective in reducing pain and disability in individuals with chronic non‐specific neck pain (CNNP). Spine SE, activating deep muscles and reducing the excessive activity of superficial muscles, has become popular to treat and prevent musculoskeletal disorders of the spine. SE may realize benefits by improving strength, endurance and coordination of the spine‐stabilizing muscles in individuals with neck pain [54].

Despite the effectiveness of SE, these exercises do not have a significant effect on vertical alignment, and connection and coordination of movements of the cervical‐thoracic‐lumbar‐pelvic chain. This is potentially important as the thoracic‐lumbar‐pelvic chain provides a solid base for the cervical vertebrae. If the thoracic chain or the lumbar‐pelvic function is unstable, the cervical motor chain undergoes a change in function to compensate [59]. Indeed, Jull et al. reported that the instability of the lumbar‐pelvic chain deactivates the deep stabilizers such as longus colli and longus capitis, and over‐activates superficial muscles such as sternocleidomastoid and anterior scalene [36], that in the end often leads to a forward head position along with an increase in the thoracic bulge and cranio‐cervical arch [59].

To address misalignment of the cervical‐thoracic‐lumbar‐pelvic chain DNS was introduced that simultaneously activates the central muscle chain, deep neck flexor, lumbosacral and extensor muscles, as well as the diaphragm and pelvic surface muscles in the entire spine [18]. However, there have been conflicting results reported regarding the effectiveness of SE and DNS in improving symptoms of neck pain [26, 28]. Therefore, the aim of this study was to assess the effects of SE and DNS on outcomes for individuals with CNNP. It was hypothesized that 6 weeks of either intervention leads to improvement in pain intensity, reduced forward head angle (FHA) and reduced electrical activity of muscle activation. The second hypothesis was that DNS is more effective compared to SE.

## METHODS

### Participants

**Figure 1:**
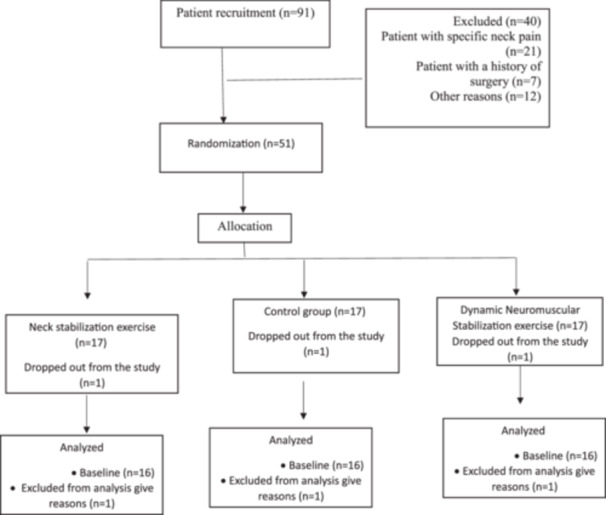
Flow diagram of participant's recruitment.

This was a retrospective study (IRCT20200622047888N3) with a pre‐test and post‐test design with two intervention groups and one control group. This study was carried out between January and July 2021 after obtaining ethical approval from the Ethics Committee of Hormozgan University of Medical Sciences (Reference number IR.HUMS.RES.1402.219). Informed written consent was obtained from each patient before participation in this study. A total of 45 women with CNNP were selected from among the administrative staff of Hormozgan University, Bandarabbas and other offices in Bandarabbas through online advertisements (Figure 1). The number of participants was based on G*Power software (effect size [ES] = 0.40, alpha level = 0.05, power = 0.80, version 3.1.9.2). We focused on women as the prevalence of neck pain is higher in women than in men [15, 33]. In addition, the number of samples was also based on previous studies [1]. All experiments were performed in accordance with the Declaration of Helsinki.

The criteria for entering the study included having persistent neck pain for more than 3 months, using a computer for at least 4 h a day, obtaining a minimum score of visual analogue scale = 3 one week before the pre‐tests. Participants also had to refrain from the use of pain medication for the duration of the study. The exclusion criteria were: having a history of neurological or psychological illness, fibromyalgia, spine surgery, heart disease or high blood pressure, neck arthritis, cervical disc herniation, spinal canal stenosis in this area, pregnancy and use of physiotherapy or drug interventions in the past 3 months. Patients who met the criteria for entering the study, after the non‐specific diagnosis of neck pain by a specialist physician, were entered into the study and were studied randomly in one of three groups: (1) SE; (2) dynamic neuromuscular stabilization (DNS); and (3) control. Groups 1 and 2 received a 6‐week intervention, while the control group did not receive any intervention. Patients were evaluated at the beginning and at the end of 6 weeks of therapeutic intervention.

Random allocation software was used to randomly assign subjects to intervention and control groups. The groups did not receive any information about the existence of a parallel group.

### Procedures

In the present study, the experimental groups received the SE and DNS intervention programmes for 6 weeks, three sessions a week on even days, while the control group did not receive the intervention. The SE interventions programme was performed for approximately 55 min per session (Table 1), which was done in the following way.

**Table 1:** Neck stabilization exercise.

Resistance exercise	Repetitions per week	Number of repetitions	Repetition time	Rest between repetitions	Rest between movements	Total time
Head resistance in forward and side bending' and turning left and right using an elastic rope	Three days per week	3 repetitions	15 s	30 s	1 min	30–40 s
Chin tuck in a sitting position on the Swissball and at the same time flexing the shoulder 90–120° and then lifting the opposite leg.						
Controlling the Swissball with the back of the head and forehead, and simultaneously shoulder abduction up to 90° with an elastic band.						
Picking up the Swissball and chin tuck						

**Figure 2:**
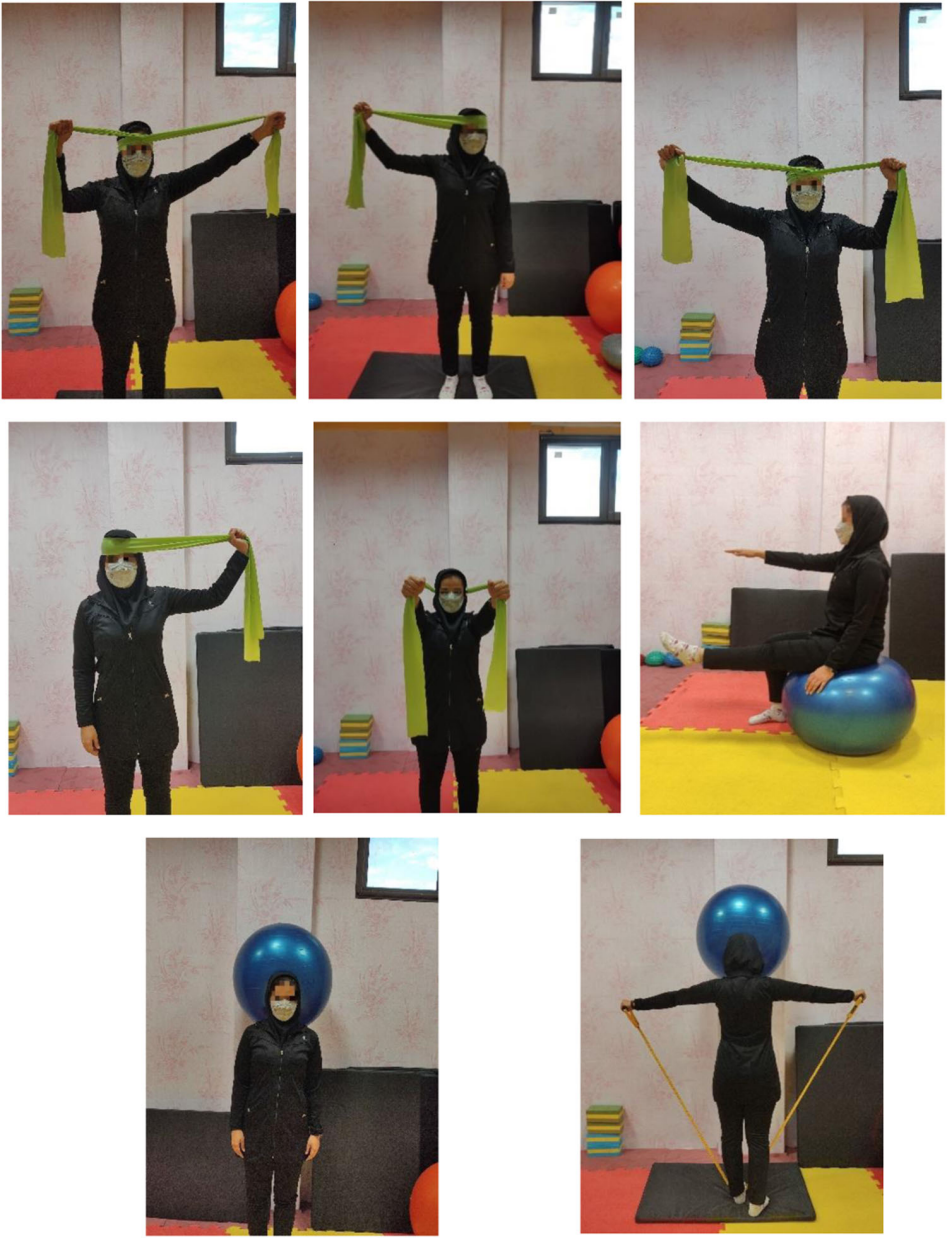
Neck stabilization exercise.

Each exercise session lasted 45–55 min and included 10 min of warm‐up, 30–40 min of specific exercises and 5 min of cooling down. The exercises included: resistance of the head against elastic stretch in different directions, control of a Swissball with the back of the head and forehead, chin tuck in a sitting position on the Swissball, three 15‐s repetitions each time (Figure 2), Rest between each set was 30 s, and 1 min between each exercise All exercises were done in groups of up to four individuals [13]. Exercises were adapted based on the principle of gradual overload; intensity, repetition and time increased from the first week based on individual characteristics (Table 1).

**Figure 3:**
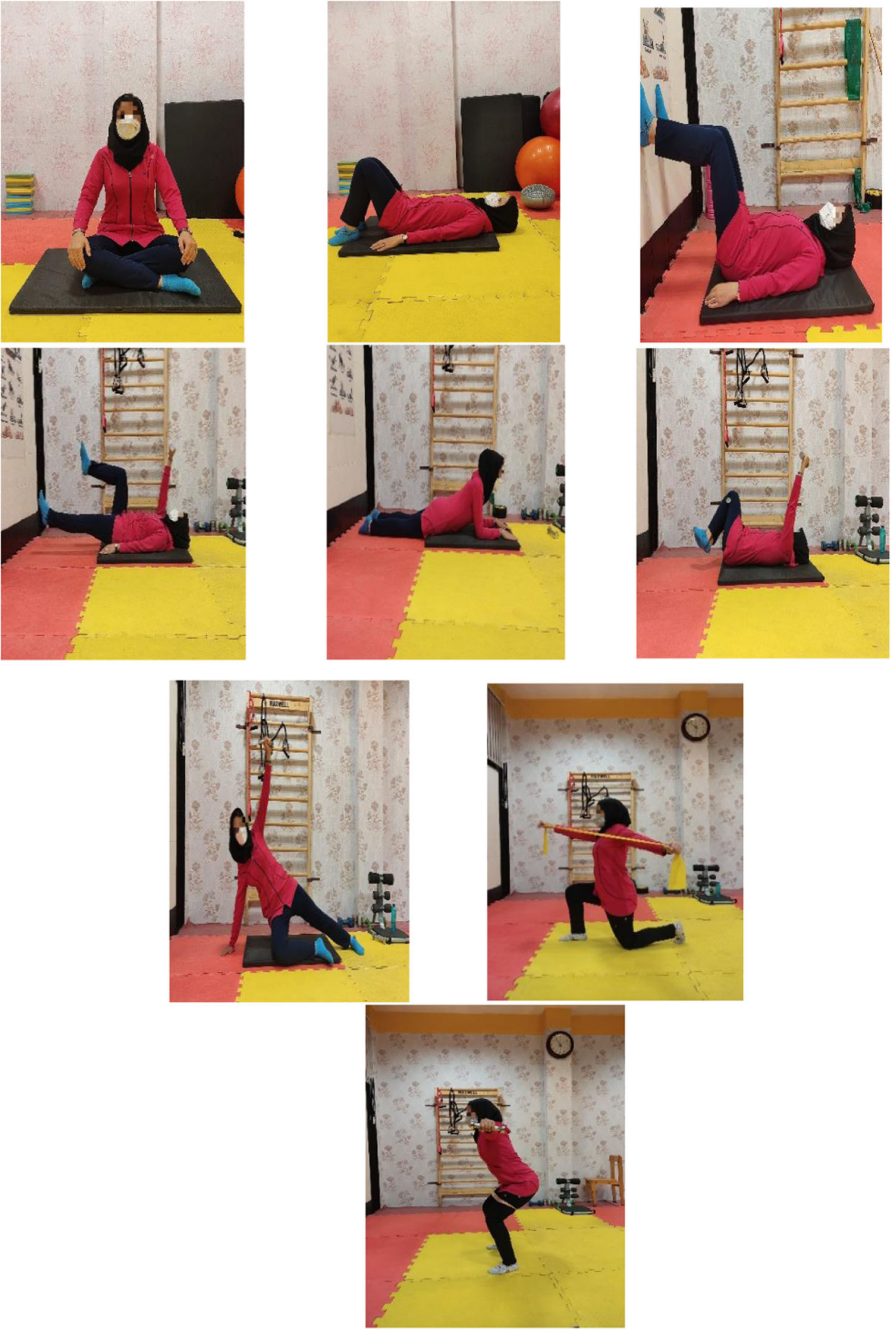
Dynamic neuromuscular stabilization exercise.

The DNS interventions programme was performed for approximately 50 min per session (Figure 3), which was done in the following way.

There was a 5‐min warm‐up, followed by 40 min of DNS movements with breathing exercises, and 5 min of cooling down. The exercises included: diaphragmatic breathing, Baby Rock (supine 90–90), Prone, Rolling, Side Lying, Oblique Sit, Tripod, Kneeling, Squat and Czech Get Up (CGU). The progress and complexity of the exercises were increased by adding a new task to the practised movement every week (a new task should not disturb diaphragmatic breathing) [47].

The control group did not receive any intervention. Although we did not prohibit them from taking medicine or doing physiotherapy, the patients confirmed that none of them had any intervention during this period. At the end of the study, they received the stabilization protocol for 6 weeks.

The pre‐ and post‐intervention measurements employed in the current research were: Numerical Pain Rating Scale (NPRS), FHA and muscle electromyography (EMG). An independent blinded investigator performed these assessments.

#### NPRS

The NPRS was used to measure the pain intensity in the neck area. Patients were asked to report the intensity of neck pain using an 11‐point scale (0 = no pain, 10 = the worst imaginable pain) [24]. The NPRS is a reliable (intraclass correlation coefficient [ICC] = 0.76, 95% confidence interval [CI], 0.51–0.87) and valid (Pearson *r* = 0.57; *p* = 0.01) tool for measuring pain intensity in patients with mechanical neck pain [14].

#### FHA

The FHA was measured by photographing the side view of the body (Nikon 5300, made in Japan). To measure this angle, the ear tragus and the spinous appendage of the seventh cervical vertebra were determined. Then the subject was asked to stand in the designated place next to the wall (at a distance of 23 cm) so that his left arm was next to the wall and the camera was at a distance of 2.65 m and at shoulder level of the person; the subject should be in a completely comfortable and natural position while looking at the hypothetical point in front of her/him. After a 5‐s pause, three sequential photos were taken ImageJ software was used to determine the FHA. This method has previously been reported to be reproducible (ICC = 0.88) [20].

#### Muscle EMG

The activation of the neck muscles (cervical erector spine [CES], sternocleidomastoid [SCM] and upper trapezius [UT]) [51] was measured in the slump and upright typing posture (the position of text typing was held 90 s with 2‐min rest between each position) using surface EMG. After skin preparation at the right side of the CES, UT and SCM to reduce skin impedance, cleaning was performed using alcohol 70%, and the skin was gently abraded using light sandpaper. Then, the surface EMG electrodes were attached 1–2.5 cm apart in parallel with both the CES, SCM and UT muscle fibres. The EMG sensors were attached around both C4 areas for the CES, slightly outward from the midline between C7 and the acromioclavicular joint for the UT and on the distal 1/3 of the muscle belly in the sternal head of the SCM. The raw EMG of the muscles was recorded at the right side (Noraxon) using a pair of Ag‐AgCl disposable surface electrodes (diameter: 20 mm; Skintact). The sampling rate of the EMG signal was set at 1000 Hz with a signal amplification gain of 1000 and common mode rejection ratio of 108 dB. The frequency bandpass filter was set to 20–500 Hz, and the location of the surface electrodes was based on the SENIAM recommendations. Maximal voluntary contraction (MVC) was also performed for amplitude normalization [11, 51, 66]. For the EMG normalization procedure, each participant performed three trials of maximum voluntary isometric contractions. Each MVC was performed for five seconds with a 2‐s rest interval. Muscle activity was tested in the sitting position for the right UT and CES muscles; the CES and SCM muscles were examined with resisted neck extension and rotation respectively, and the UT muscles were tested with resisted shoulder elevation.

## STATISTICS

The number of participants was based on G*Power software (effect size [ES] = O.40, alpha level = 0.50, power = 0.80, version 3.1.9.2) (G*Power© from the University of Dusseldorf). To allow for a dropout rate of 10% and improve final statistical power, we enroled 15 participants per group (total sample size of 45 participants). The Shapiro–Wilk test was used to assess the normality of data. A two‐way group (between factor three levels) × time (within factor: pre vs. post) ANOVA with repeated measures was used to compare differences between groups and the impact of the 6‐week intervention. Bonferroni‐adjusted post hoc tests were performed where needed. All variables are reported as mean ± standard deviation, and effect size, confidence interval were calculated to provide a measure of clinical meaningfulness. The significance level will be set at *p* < 0.05.

## RESULTS

There was no significant difference in the demographic and clinical variables between the three groups at baseline (*p* > 0.05; Table 2).

**Table 2:** Demographic data and baseline values of patients with chronic non‐specific neck pain.

Characteristic	SE	DNS	Control	*p* value
Body mass (kg)	70.3 ± 8.7	70.5 ± 12.8	73.1 ± 15.4	0.43
Age (years)	42.5 ± 5.0	39.7 ± 7.0	43.1 ± 7.2	0.23
Height (cm)	164 ± 4	161 ± 4	162 ± 6	0.56
Pain duration (year)	4.10 ± 2.03	3.92 ± 3.17	3.03 ± 1.80	0.41
Work time (h)	6.21 ± 1.84	7.35 ± 1.73	6.28 ± 2.61	0.48

Abbreviations: DNS, dynamic neuromuscular stabilization; SE, stabilization exercise.

### Treatment effects

The main effects and group × time interactions are presented in Tables 3 and 4. Significant group × time interactions were found for NPRS (*F* (2,39) = 17.61, *p* = 0.001, partial *η*² = 0.475) (Table 3), FHP (*F* (2,39) = 5.509, *p* = 0.008, partial *η*² = 0.220) (Table 3), amount of activity in the CES muscle (*F* (2,39) = 5.31, *p* = 0.009, partial *η*² = 0.214), amount of activity in the UT muscle (*F* (2,39) = 5.41, *p* = 0.008, partial *η*² = 0.217).

**Table 3:** NPRS and FHP before and after the 6‐week interventions.

					Within‐subject effect		Between‐groups	
Outcomes	Group	Baseline (mean ± SD)	6 weeks (mean ± SD)	Δ Relative to baseline (%)	ES (95% CI)	*p*	*p*	Interaction effect (group × time)
NPRS	SE	5.18 ± 1.52	2.75 ± 1.77	46.9 ↓^e^	0.59 (2.21–3.28)	0.01 d	*p* = 0.01^b^ *p* = 0.01^a^	*p* = 0.01^d^
	DNS	4.75 ± 1.37	1.85 ± 1.54	61 ↓^d^	0.70 (1.38–2.31)	0.01 d		
	Control	4.90 ± 1.87	4.83 ± 2.03	2.2 ↓^e^	0.17 (4.22–5.43)	0.86		
FHP	SE	41.34 ± 5.47	39.15 ± 5.62	5.29 ↓^e^	0.74 (39.6–43.0)	0.03 d	*p* = 0.11	*p* = 0.01^d^
	DNS	44.82 ± 4.62	41.84 ± 4.48	6.6 ↓^e^	0.31 (40.5–43.2)	0.01 d		
	Control	42.93 ± 6.15	43.12 ± 4.72	0.4 ↑^e^	−0.01 (41.7–44.5)	0.83		

*Note*: ^a^Significant difference between NSE or ^b^Dynamic neuromuscular SE and control group. ^c^Denotes significant within‐group difference from baseline to post 6‐week treatment period. ^d^Significant group × time interaction. ^e^Per cent change (↓decrease, ↑increase).

Abbreviations: CI, confidence interval; DNS, dynamic neuromuscular stability; ES, effect size; FHP, forward head position; NPRS, Numerical Pain Rating Scale; NSE, neck stabilization exercise; SE, stabilization exercise.

**Table 4:** The muscles activity before and after the 6‐week interventions.

						Within‐subject effect		Between‐groups	
Outcomes	Typing posture	Group	Baseline (mean ± SD)	6 weeks (mean ± SD)	Δ Relative to baseline (%)	ES (95% CI)	*p*	*p*	Interaction effect (group × time)
EMG CES	Slump	SE	8.51 ± 4.64	5.90 ± 2.27	30.6 ↓^e^	0.71 (−1.79 to 0.36)	0.02^c^	*p* = 0.04^b^	*p* = 0.01^d^
		DNS	10.44 ± 8.57	5.28 ± 3.60	49.4 ↓^e^	0.78 (−1.87 to 0.30)	0.02^c^		
		Control	7.24 ± 3.48	8.40 ± 3.98	16.00 ↑^e^	0.31 (−0.74 to 1.36)	0.052		
	Upright	SE	6.22 ± 3.57	5.86 ± 4.04	5.7 ↓^e^	0.09 (−1.14 to 0.95)	0.80	*p* = 0.74	*p* = 0.92
		DNS	7.56 ± 9.32	8.81 ± 16.93	16.5 ↑^e^	0.09 (−0.95 to 1.14)	0.80		
		Control	6.93 ± 4.09	7.84 ± 3.93	13.1 ↑^e^	0.22 (−0.82 to 1.27)	0.04^c^		
EMG SCM	Slump	SE	4.28 ± 1.95	3.98 ± 3.85	7.0 ↓^e^	0.09 (−1.14 to 0.95)	0.76	*p* = 0.37	*p* = 0.08
		DNS	4.67 ± 2.09	2.59 ± 1.79	44.5 ↓^e^	0.99 (−2.18 to 0.05)	0.01^c^		
		Control	3.14 ± 1.48	3.10 ± 1.59	1.2 ↓^e^	0.02 (−1.07 to 1.02)	0.91		
	Upright	SE	4.11 ± 2.23	6.43 ± 14.56	56.4 ↑^e^	0.22 (−0.82 to 1.27)	0.56	*p* = 0.67	*p* = 0.82
		DNS	4.21 ± 2.17	5.23 ± 6.44	24.2 ↑^e^	0.21 (−0.83 to 1.26)	0.51		
		Control	3.13 ± 1.71	3.34 ± 1.05	6.7 ↑^e^	0.14 (−0.90 to 1.19)	0.49		
EMG UT	Slump	SE	7.02 ± 2.97	4.77 ± 1.85	32.0 ↓^e^	0.90 (−2.01 to 0.19)	0.01^c^	*p* = 0.62	*p* = 0.00^d^
		DNS	5.81 ± 3.50	5.45 ± 1.36	6.1 ↓^e^	0.13 (−1.18 to 0.91)	0.68		
		Control	4.25 ± 2.24	5.35 ± 2.54	25.8 ↓^e^	0.45 (−0.60 to 1.52)	0.43		
	Upright	SE	4.86 ± 2.81	4.56 ± 1.91	6.1 ↓^e^	0.12 (−1.17 to 0.92)	0.99	*p* = 0.37	*p* = 0.47
		DNS	5.35 ± 3.22	8.74 ± 12.66	63.3 ↑^e^	0.36 (−0.68 to 1.42)	0.33		
		Control	4.85 ± 1.96	5.85 ± 2.23	20.61 ↑^e^	0.47 (−0.58 to 1.53)	0.05		

*Note*: ^a^Significant difference between NSE or ^b^Dynamic neuromuscular SE and control group. ^c^Denotes significant within‐group difference from baseline to post 6‐week treatment period. ^d^Significant group × time interaction. ^e^Per cent change (↓decrease, ↑increase).

Abbreviations: CES, cervical erector spinae; CI, confidence interval; DNS, dynamic neuromuscular stability; ES, effect size; NSE, neck stabilization exercise; SCM, sternocleidomastoid; SE, stabilization exercise; UT, upper trapezius.

### NPRS

**Figure 4:**
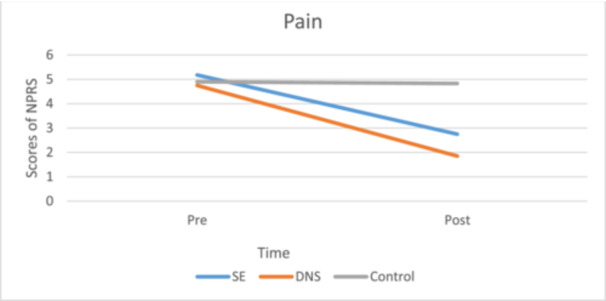
Overall scores of NPRS before and after the intervention. DNS, dynamic neuromuscular stability; NPRS, Numerical Pain Rating Scale; SE, stabilization exercise.

Six weeks of SE resulted in a 46.9% (ES [95% CI] = 0.59 [2.21–3.28], *p* < 0.01) and DNS in a 61.0% (ES [95% CI] = 0.70 [1.38–2.31], *p* < 0.01) reduction in NPRS score, but in the control groups, there was no significant improvement (*p* = 0.86). There was no significant difference in the reduction in NPRS score between the two intervention groups (Figure 4).

### Forward head

Six weeks of SE resulted in a 5.29% (ES [95% CI] = 0.74 [39.62–42.98], *p* < 0.03) and DNS in a 31.0% (ES [95% CI] = 0.31 [40.49–43.18], *p* < 0.01) reduction in FHP had decreased, but in the control groups, there was no significant improvement (*p* = 0.83). There was a significant difference in the reduction in FHP angle score between the two intervention groups. Based on the above information, the reduction in FHA is greater in the dynamic stability group (Table 3).

### Muscle activity in slump posture

**Figure 5:**
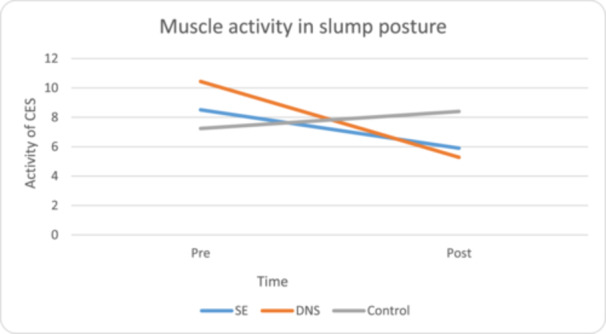
The EMG of CES in slump posture before and after the intervention. CES, cervical erector spine; DNS, dynamic neuromuscular stability; EMG, electromyography; SE, stabilization exercise.

Six weeks of SE resulted in a reduced CES (30.6%; ES [95% CI] = 0.71 [−1.79 to 0.36]; *p* = 0.02) and UT (32.0%; ES [95% CI] = 0.90 [−2.01 to 0.19]; *p* = 0.01), but no significant change in SCM (*p* = 0.76) activity in slump typing posture. Six weeks of DNS resulted in reduced activity of CES (49.4%; ES [95% CI] = 0.78 [−1.87 to 0.30]; *p* = 0.02) and SCM (44.5%; ES [95% CI] = 0.99 [−2.18 to 0.05]; *p* = 0.01), but not UT (*p* = 0.68) in slump typing posture were significantly reduced (Table 4), but in the control groups there was no significant improvement (Table 4). There was a significant difference in the reduction in CES and SCM, between the two intervention groups. Based on the above information, the reduction in CES and SCM is greater in the dynamic stability group (Figure 5).

### Muscle activity in upright posture

Neither SE nor DNS resulted in a significant reduction in muscle activity in upright posture (Table 4).

## DISCUSSION

This study investigated the effect of SE or DNS on patients with CNSNP. The main observation of the study was that SE and DNS led to significant improvement in pain intensity, decreased FHP, and normalization of muscle activity after 6 weeks in patients with CNSNP.

Previous studies reported improvements in FHP after stretching and strengthening exercises [23, 42, 43, 46, 60]. Consistent with these findings, this study found that 6 weeks of ES or DNS exercise also improved FHP.

The DNS exercise did not directly involve the movement of the neck or shoulder area, but perhaps indirectly influenced the structure of the neck via increasing intraperitoneal pressure through diaphragm‐assisted breathing that stabilizes the trunk. In addition, stability may have been enhanced through the integrated spinal stabilizing system, which activates not only the deep neck flexor muscles, the erector spinae, the upper and lower back muscle, and the lumbar erector, but also the diaphragm, pelvic floor muscles and abdominal muscles [8, 56], all important for stability.

Improved muscle coordination and stabilization of the centre of the trunk after DNS may also contribute to pain reduction [53]. In fact, one of the main causes of chronic pain is low muscle endurance, which leads to early fatigue and increased pressure on the spine, where DNS may delay the onset of muscle fatigue, and hence pain, through improved trunk stabilization [6]. In line with this, Emami et al. observed that central SEs in individuals with back pain both increased the endurance of the trunk muscles and reduced pain [7]. Thus, central stabilization and improvement of muscle fatigue resistance are instrumental in pain reduction, where stability of the spine will result in a diminished pressure exerted on the intervertebral discs [21], Further corroborating this idea is a systematic review of four cross‐sectional studies (including two high‐quality studies) that showed a significant relationship between an individual's posture with neck pain [5] and FHP [61].

These observations indicate that improper posture can cause neck pain that can be alleviated with DNS via reduction of the FHA [5, 61]. This improvement in FHP, in turn, can be due to a decrease in the cervical erector spinae muscle activity [2].

The reduction in neck pain following SE is consistent with previous studies [22, 28, 39]. Falla et al. [22] reported that people with neck pain have an impaired ability to stabilise the head position, which could be restored by regular exercise that improves the strength and endurance of muscles. This improved ability to stabilize the head reduces the pressure on the neck. Therefore, therapeutic exercise is useful to prevent musculoskeletal complications by strengthening muscles, preventing repetitive movements and increasing blood supply (delivering oxygen and nutrients to muscle cells) [22].

Other causes of pain reduction and subsequently disability reduction in our study may be the reduction of gamma efferent nerve activity and a better regulation of muscle tone [38]. In fact, SEs directly activate the deep flexor muscles of the neck, which have many muscle spindles. The repeated contractions in SEs may improve the function of the spindles and lead to enhanced proprioception. Another possible mechanism of pain improvement following SEs can be improved coordination in the recruitment of superficial and deep muscles [50].

As an indication of an altered recruitment strategy, we observed here that DNS and ES resulted in a decreased activation of the ES, UT and SCM muscles, superficial muscles that play a primary role in neck position and stability, during slump but not upright posture [9]. Similar to our observation, it has been shown with EMG that the increased UT recruitment in individuals with neck pain can return to optimal muscle activity levels after scapular postural correction exercise [65]. Damage to afferent receptors within the muscles or soft tissue of the spine could impair the magnitude and timing of somatosensory feedback from the muscle to the central nervous system and, in turn, delay the reflex response [32]. Therefore, the reduced activation of the UT, SCM and CES muscles during slump posture after the DNS and ES may result in reduced pain and spinal neuronal excitability [31]. This then may stimulate positive alterations in posture and proper firing and recruitment patterns of muscles, which are considered necessary for normal neck and scapular orientation [44]. In line with our observation, the literature shows that the reduction of muscle activation after an exercise intervention is effective at decreasing the muscle activities of the UT, CES, sternal head of the SCM and anterior scalenes when performing a given task [12]. The mechanisms involved in decreasing muscle activation in the strength endurance exercise group might be improved muscle coordination [48], which will also delay the onset of muscular fatigue. In the exercise group, the positive effect might have accrued from a combination of positive alterations in neuromuscular efficiency and cervical motor control strategies, resulting in positive changes in the superficial muscles.

Studies have reported the effectiveness of training in improving posture in patients suffering from chronic neck pain [4, 29]. The results of the present study are consistent with these studies, demonstrating that exercise interventions could result in positive and significant alterations in posture in patients with chronic neck pain. FHP and PSP are related to shortening of the UT, posterior cervical extensor muscles (suboccipital, semispinalis and splenii), SCM and levator scapulae muscles and weakness of the deep cervical flexor [45]. According to previous research, chin‐tuck alone is not a large enough stimulus to realize improvements in FHP and reductions in pain [10, 45]. Therefore, researchers have tried to combine chin‐tuck with some other endurance and strength exercises to enhance the range of motion, muscle endurance, and strength of the neck. The mechanisms involved in the positive alteration of posture in the exercise group might have accrued from a combination of improvement in neuromuscular efficiency [45], cervical motor control strategies improving the deep cervical flexor muscle [34], and correction of scapular alignment of depression, downward rotation, or abduction (internal rotation) [57]. Also, this study has shown a positive alteration in posture. The alteration might be a result of the combined mechanisms of both minimizing the activation of superficial muscles and strengthening the weakened muscles during arm movements [58], decreasing compressive forces on the cervical apophyseal joints and posterior part of the vertebra, and changes in connective tissue length and strength which resulted from bad posture [62].

The greater effectiveness of DNS compared to neck stabilization exercise (NSE) in this study may be related to the fact that dynamic stability exercises depend on muscle stability and coordination of both local and global muscles to ensure proper joint alignment in the kinetic chain. The quality of this coordination of both local and global muscles will not only improve joint performance but also have an impact on biomechanical and anatomical parameters. Furthermore, the ultimate objective of this intervention (DNS) is to maintain central control, joint stability and ideal quality of movement through automatic repetition of movements and central control. On the other hand, DNS focuses on the importance of precise timing of muscle activity and efficient coordination, as well as resistance to compressive forces in static movements [40].

This study had several limitations. First, this was a retrospective study, so its long‐term effects are unclear. In addition, we only studied women, but it is likely that the impact of ES and DNS will not be sex‐dependent. Another interesting follow‐up study may be to assess the benefits of combining SE and DNS.

## CONCLUSION

This study reports positive postural alterations, reductions in neck pain and a reduction in superficial neck muscle activation patterns in slump posture after either NSE or DNS performed three times a week for 6 weeks by patients with chronic neck pain. DNS seems to have slightly better improvements than SE. This suggests a relationship between the correction of FHP, neck pain and activation of the superficial muscles.

## AUTHOR CONTRIBUTIONS

Zahra Ataei Cheragh conceived the study and was responsible for the project administration. Maryam Mazidi, Ainollah Sakinepoor and Zahra Ataei Cheragh designed data collection and analyzed the data. Zahra Ataei Cheragh and Ainollah Sakinepoor wrote the first draft of the paper. Hans Degens critically reviewed and approved the final manuscript. All authors read and approved the final version of the manuscript.

## CONFLICT OF INTEREST STATEMENT

The authors declare no conflicts of interest.

## ETHICS STATEMENT

This study was approved by the Ethics Committee of Hormozgan University of Medical Sciences under the reference number IR.HUMS.RES.1402.219. And carried out in accordance with relevant guidelines and regulations. The study protocol was clearly explained to all participants before the data were collected. Informed consent was obtained from all participants included in the study. The study was registered with the retrospective study registry IRCT20200622047888N3.

## Data Availability

The data sets used and/or analyzed during the current study are available from the corresponding author on reasonable request.
